# Deciphering Gut Microbiome in Colorectal Cancer via Robust Learning Methods

**DOI:** 10.3390/genes16040452

**Published:** 2025-04-15

**Authors:** Huiye Han, Ying Li, Youran Qi, Stefano Mangiola, Wodan Ling

**Affiliations:** 1 Division of Biostatistics, Department of Population Health Sciences, Weill Cornell Medicine, New York, NY 10065, USA; huh4008@med.cornell.edu (H.H.); yil4013@med.cornell.edu (Y.L.); 2 Independent Researcher, New York, NY 10128, USA; youran.qi@gmail.com; 3 South Australian immunoGENomics Cancer Institute, The University of Adelaide, Adelaide, SA 5005, Australia; stefano.mangiola@adelaide.edu.au; 4 Division of Bioinformatics, Walter and Eliza Hall Institute of Medical Research, Parkville, VIC 3052, Australia

**Keywords:** colorectal cancer, gut microbiota, data integration, robust differential composition analysis, microbial signatures

## Abstract

Background: Colorectal cancer (CRC) is one of the most prevalent cancers worldwide and is closely linked to the gut microbiota. Identifying reproducible and generalizable microbial signatures holds significant potential for enhancing early detection and advancing treatment for this deadly disease. Methods: This study integrated various publicly available case-control datasets to identify microbial signatures for CRC. Alpha and beta diversity metrics were evaluated to characterize differences in gut microbial richness, evenness, and overall composition between CRC patients and healthy controls. Differential abundance analysis was conducted using ANCOM-BC and LEfSe to pinpoint individual taxa that were enriched or depleted in CRC patients. Additionally, sccomp, a Bayesian machine learning method from single-cell analysis, was adapted to provide a more robust validation of compositional differences in individual microbial markers. Results: Gut microbial richness is significantly higher in CRC patients, and overall microbiome composition differs significantly between CRC patients and healthy controls. Several taxa, such as *Fusobacterium* and *Peptostreptococcus*, are enriched in CRC patients, while others, including *Anaerostipes*, are depleted. The microbial signatures identified from the integrated data are reproducible and generalizable, with many aligning with findings from previous studies. Furthermore, the use of sccomp enhanced the precision of individual microbial marker identification. Conclusions: Biologically, the microbial signatures identified from the integrated data improve our understanding of the gut microbiota’s role in CRC pathogenesis and may contribute to the development of translational targets and microbiota-based therapies. Methodologically, this study demonstrates the effectiveness of adapting robust techniques from single-cell research to improve the precision of microbial marker discovery.

## 1. Introduction

Colorectal cancer (CRC) is one of the most prevalent cancers worldwide, causing a significant proportion of cancer-related morbidity and mortality [1,2]. Research has shown that the gut microbiota plays a pivotal role in both CRC development and suppression. A particular example is that the dysbiosis (imbalanced microbial composition) contributes to the initiation and progression of CRC [3,4,5,6,7]. Therefore, identifying microbial signatures that are associated with CRC holds great potential to transform the diagnosis, prognosis, and therapeutics of CRC, making it a key area of computational CRC research.

In this study, we aim to decipher microbial markers in CRC at both the community and individual levels. At the community level, richness and evenness of microbial profiles and alternations of overall microbial compositions were investigated. At the individual level, the target is to identify differentially abundant taxa between CRC patients and healthy controls. To uncover signals that are unattainable within a single study (improving sensitivity) and avoid spurious findings due to biases in individual studies (enhancing robustness), we integrated microbiome datasets from various publicly available CRC studies. Given the typically small sample sizes in CRC research, this integration is essential for making reliable discoveries about microbiome–CRC relationships [8]. The conditional quantile regression (ConQuR) method [9] was used to mitigate between-study artifacts during the integration.

Facilitated by the resulting large-scale microbiome data, alpha and beta diversities were examined to understand the microbial community shifts between CRC and healthy individuals. To identify specific taxa driving these shifts, differential abundance analysis (DAA) was performed. DAA presents unique challenges due to the inherent characteristics of microbiome data, including high dispersion, sparsity (excessive zero abundances), and compositionality [10]. To address these challenges, we employed several robust learning methods. In addition to the well-received linear discriminant analysis effect size (LEfSe) [11] and analysis of composition of microbiomes with bias correction (ANCOM-BC) [12] that are tailored for microbiome data, we also adapted sccomp [13], which was originally developed for single-cell data. The sccomp method provides a Bayesian framework for robust differential composition and variability analysis. It effectively captures the mean–variability relationship of relative abundance, thus enhancing the reliability of microbial signature identification.

Our identified microbial signatures for CRC, at both the community and individual levels, validate and expand upon existing findings in biomedical literature. Additionally, detailed comparisons of the DAA methods highlight the value of sccomp in improving and complementing microbiome-specific methodologies. This sheds light on the potential of adapting robust learning methods originally designed for single-cell analysis to advance microbiome research.

## 2. Materials and Methods

### 2.1. Data Source and Processing

The data for this study were obtained from the MicrobiomeHD database [14]. MicrobiomeHD is a standardized repository that collects human gut microbiome data related to health and disease [15]. This database includes publicly available 16S rRNA sequencing data from published case-control studies, along with associated patient metadata. To ensure data quality and reliability of the analysis, a dataset included in the database is required to meet the following criteria: it has publicly available raw sequencing data in FASTQ format, it has metadata containing case and control labels, and it has a minimum of 15 cases.

The raw 16S rRNA gene sequencing data in the MicrobiomeHD database were pre-processed using Alm lab’s in-house 16S processing pipeline [16]. This pipeline can filter to remove low-quality sequences and cluster sequences into operational taxonomic units (OTUs) by various OTU calling methods, such as Mothur [17], QIIME [18], etc. Taxonomy was assigned using reference databases, such as SILVA [19], RDP [20], and BLAST [21]. The data were then agglomerated to the genus level, which is the lowest taxonomic resolution that 16S rRNA sequencing can reliably identify. We selected all five of the CRC studies from the database (the Baxter, Xiang, Zhao, Zackular, and Zeller studies [22,23,24,25,26]), covering a total of 130 CRC patients and 200 healthy controls.

Given the differential experimental designs, sample handling protocols, and bioinformatic processing methods of the five studies (Table A1), simply combining their datasets could bring in unwanted technical heterogeneity, leading to false discoveries and obscuring true biological signals [27]. To remove these artifacts while preserving biological signals, we applied ConQuR to the taxonomic counts. In particular, ConQuR employs a conditional quantile regression framework to match the conditional distributions of taxon abundance across studies given important biological factors. Thus, it effectively regresses out artifacts while maintaining the biological effects of interest. This procedure was guided by the MicrobiOme Studies Analytical Integration and Correction (MOSAIC) pipeline, which systematically evaluates microbiome data integration strategies, including ConQuR, for merging a given collection of microbiome datasets [28].

To avoid potential false findings from rare taxa, genera present in fewer than 1% of all samples were excluded, leaving 63 genera for analysis. Finally, to ensure comparability across samples with different sequencing depths, the taxonomic counts were normalized to relative abundances (count divided by sequencing depth within each sample).

### 2.2. Statistical Methods

Leveraging the processed data, we aimed to learn the microbial signatures that can differentiate CRC patients from healthy controls.

For the community-level analysis, we first examined alpha diversity, which measures the richness and evenness of taxa within a sample. Two metrics were used, richness—the number of unique taxa in a sample, and Shannon index—an entropy-based measure computed based on relative abundances accounting for both richness and evenness. The differences in alpha diversity between the CRC and control groups were assessed using the Wilcoxon rank sum test.

Next, we evaluated beta diversity using two distance metrics—Jaccard and Aitchison distances. For each pair of samples, the Jaccard distance is the the ratio of shared taxa to the total number of taxa present in the two samples, whereas the Aitchison distance is the Euclidean distance between the two profiles of centered log-ratio (CLR) [29] transformed relative abundances (the zero abundances of 51 genera that are present in more than 20% of all samples were imputed by the geometric Bayesian multiplicative replacement [30] method before CLR transformation). These two distances reflect differences in microbial composition in terms of presence–absence and relative abundance, respectively. Principal coordinate analysis (PCoA) [31] was utilized to visualize the differences in beta diversity between the CRC and healthy groups, with statistical significance assessed by the permutational multivariate analysis of variance (PERMANOVA) [32] and microbiome regression-based kernel association test (MiRKAT) [33].

For the individual-level analysis that aims to identify differentially abundant taxa between the CRC and control groups, we first employed two widely used methods in the field, LEfSe and ANCOM-BC (Table A2). LEfSe uses robust non-parametric tests (Kruskal–Wallis and Wilcoxon rank sum tests) and linear discriminant analysis (LDA) [11]. It effectively integrates tests for statistical significance with additional tests encoding biological relevance, and can quantify effect sizes of the identified taxa. On the other hand, ANCOM-BC excels in robustly addressing the compositional bias of microbiome data. It accounts for sampling fraction by introducing a sample-specific offset term in linear regression and corrects the bias introduced by differences in the sampling fractions. Therefore, it improves the reliability in comparing the various taxa, the precision in estimating differential abundances, and the control of false discoveries. For both methods, to control the false discovery rate (FDR) for multiple comparisons across taxa, the Benjamini–Hochberg procedure was applied to adjust the *p*-values. An adjusted *p*-value (*q*-value) less than 0.05 was considered statistically significant in this study.

To further enhance the robustness of DAA, we adapted sccomp (Table A2), a Bayesian framework originally developed for analyzing single-cell composition and variability. This framework employs a sum-constrained beta-binomial model to better account for sparsity, dispersion, and compositionality, so it is particularly suitable for microbiome data. Additionally, this method can capture the inherent mean–variability relationship of microbial abundances. Note that, unlike the bimodal mean–variability relationship observed in single-cell data, microbiome data typically exhibit a unimodal pattern, which was adopted in our analysis. The Bayesian inference leverages prior information estimated from 18 previously analyzed single-cell RNA sequencing, CyTOF, and microbiome datasets. It then uses the Hamiltonian Monte Carlo (HMC) [34] method to sample posterior probabilities. The probability of the null hypothesis (no difference) is obtained by estimating the posterior probability of the contrast being larger or smaller than a fold-change threshold of 0.2. The *q*-value is obtained by sorting the probability of the null hypothesis in ascending order and calculating the cumulative average, as proposed by Stephens [35]. Moreover, outlier detection is iteratively executed by fitting the model and calculating the 95% credible intervals for each data point to identify outliers for exclusion. This process includes refitting the model without removing outliers and adjusting the posterior predictive distribution to improve detection accuracy. Overall, this adapted approach enhances DAA by reducing false positives and improving the precision of detection.

## 3. Results

### 3.1. Gut Microbial Diversity in Colorectal Cancer

#### 3.1.1. Differences in Alpha Diversity Between the CRC and Healthy Groups

**Figure 1:**
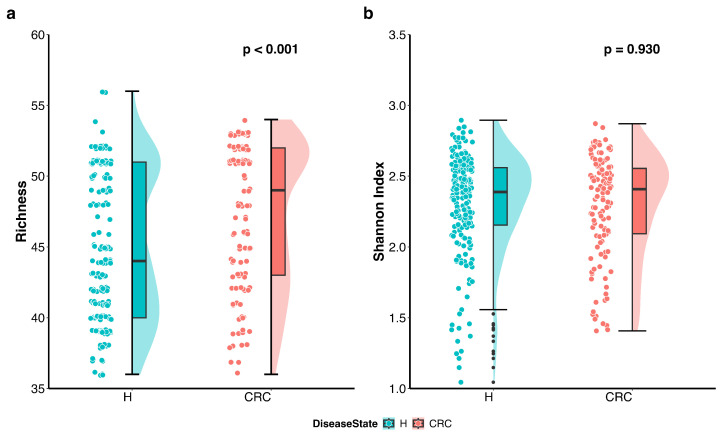
Differences in alpha diversity between colorectal cancer patients (CRC) and healthy controls (H). Violin plots with individual data points display the alpha diversity distribution in each group. (**a**) Richness in CRC patients is significantly higher than that in healthy controls (*p* < 0.001 by the Wilcoxon rank sum test). (**b**) Shannon index accounting for both richness and evenness is not significantly different between CRC patients and healthy controls (*p* = 0.930 by the Wilcoxon rank sum test).

We first visualized the differences in richness and Shannon index between the CRC patients and healthy controls using violin plots with individual data points displayed (Figure 1). The plots indicate that richness is higher in the CRC group, whereas the Shannon index accounting for both richness and evenness does not differ between the groups. Further analysis using the Wilcoxon rank sum test quantitatively confirmed that richness is significantly higher in CRC patients compared to healthy controls (*p*-value < 0.001), while the difference in Shannon index is not statistically significant (*p*-value = 0.93).

These findings suggest that CRC patients harbor a greater number of distinct microbes; however, the overall proportional distribution of various taxa within the microbial community remains unchanged compared to healthy individuals.

#### 3.1.2. Differences in Beta Diversity Between the CRC and Healthy Groups

**Figure 2:**
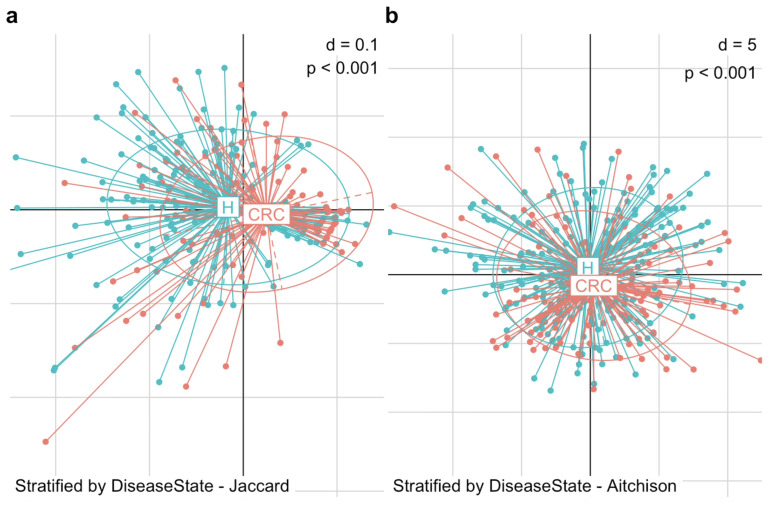
Differences in beta diversity between colorectal cancer patients (CRC) and healthy controls (H). PCoA plots display the dissimilarity in gut microbiome composition between the two groups. (**a**) PCoA plot based on Jaccard distance shows a significant structural difference in microbial community between CRC patients and healthy controls in terms of presence-absence (MiRKAT *p* < 0.001). (**b**) PCoA based on Aitchison distance shows a significant structural difference in microbial community between CRC patients and healthy controls in terms of abundance (MiRKAT *p* < 0.001).

We began by visualizing the microbiome composition using PCoA plots based on Jaccard and Aitchison distances (Figure 2). In the PCoA plot, each point represents an individual subject, while each ellipse denotes a group—the CRC or control group. The centroid of an ellipse indicates the group mean, and the ellipse itself encompasses the 95% percentile of points, reflecting the group’s dispersion. The plots reveal a substantial difference in beta diversity between CRC patients and healthy controls, in terms of the mean and dispersion of their microbial communities. To statistically assess this difference, we applied PERMANOVA and MiRKAT, both of which confirmed a significant distinction in gut microbiome composition between the two groups. This difference was evident in both the microbial presence–absence (Jaccard distance: PERMANOVA *p* = 0.001, MiRKAT *p* < 0.001) and abundance (Aitchison distance: PERMANOVA *p* = 0.001, MiRKAT *p* < 0.001).

This structural reconfiguration of the gut microbiota in CRC patients compared to healthy individuals provides a foundation for further investigation into the microbiome’s role in the progression and suppression of CRC.

### 3.2. Differentially Abundant Taxa in Colorectal Cancer

#### 3.2.1. Exploratory Analysis

Next, we aimed to identify the specific taxa driving the community shifts confirmed in Section 3.1. Before conducting a formal DAA, we visually explored the taxa to narrow down potential targets.

**Figure 3:**
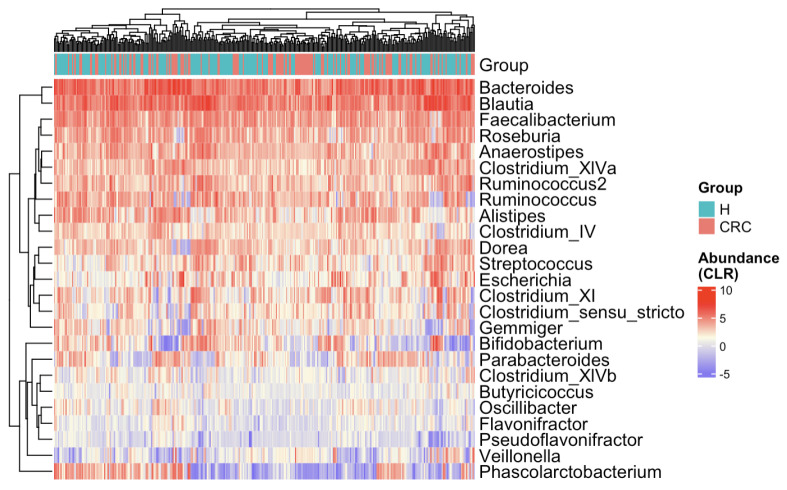
Heatmap of CLR-transformed abundances of the top 25 most common bacterial taxa. Each row (*y*-axis) represents a taxon, and each column (*x*-axis) corresponds to a subject. CRC indicates colorectal cancer patients and H indicates healthy controls. The color intensity represents the CLR-transformed abundance of each taxon. Subjects are hierarchically clustered based on their gut microbial profiles, with no distinct separation between CRC patients and healthy controls.

**Figure A1:**
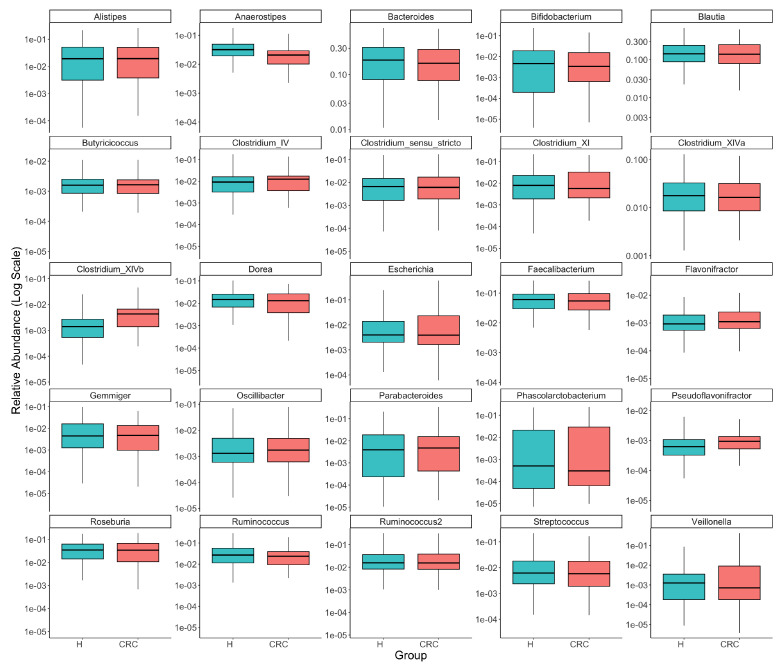
Comparison of the top 25 most common taxa’s relative abundances between colorectal cancer patients (CRC) and healthy controls (H). Each panel presents one taxon, with boxplots illustrating the distributions of its relative abundance in the CRC and healthy groups and the *y*-axis representing log-transformed relative abundance (we added 0.5 to all counts before taking log of relative abundances to avoid log of zeros). The visual exploration highlights the differential abundances of several taxa, such as *Anaerostipes*, *Clostridium_XIVb*, and *Pseudoflavonifractor*, between the CRC and healthy groups.

The heatmap (Figure 3) presents CLR-transformed abundances of the top 25 most common taxa across all subjects and clusters individuals based on their microbial profiles [36,37,38]. While there is no distinct separation between the CRC and control groups, some subtle trends emerge. For example, *Bifidobacterium* appears depleted in CRC patients, whereas *Escherichia* and *Clostridium* show relative enrichment. To further explore the marginal differences, we compared the 25 taxa’s relative abundances between the CRC and healthy groups via boxplots (Figure A1). Several taxa exhibit notable shifts—*Anaerostipes* is depleted in CRC patients, while *Clostridium_XIVb* and *Pseudoflavonifractor* appear enriched in the CRC group.

These exploratory results support the microbial community shifts from healthy individuals to CRC patients. They also highlight several genera that may play a role in either promoting or inhibiting CRC progression, warranting further DAA in the next subsection and biological investigation for confirmation.

#### 3.2.2. Differential Abundance Analysis

**Figure 4:**
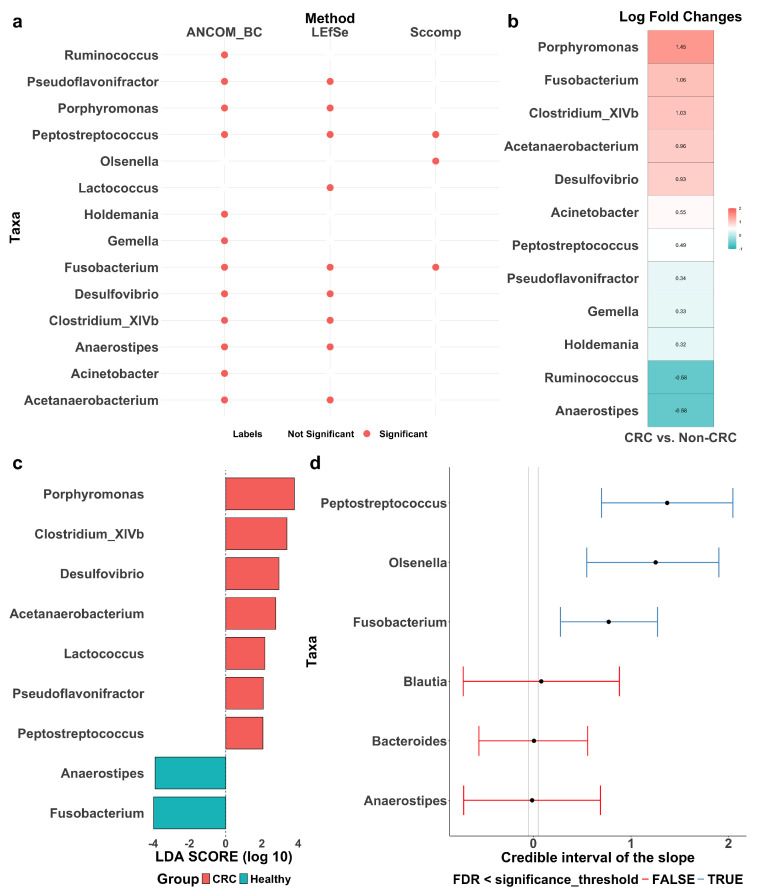
Differentially abundant taxa between colorectal cancer patients (CRC) and healthy controls (Healthy or Non-CRC). (**a**) Summary of differentially abundant taxa identified by ANCOM-BC, LEfSe, and sccomp, with red dots indicating identified taxa. (**b**) Log fold change (LFC) by ANCOM-BC, quantifying effect sizes of the identified taxa, with positive values reflecting enrichment in CRC patients. (**c**) Discriminant analysis (LDA) score by LEfSe, quantifying effect sizes of the identified taxa, with positive values reflecting enrichment in CRC patients. (**d**) Differential composition analysis results by sccomp. Error bars represent 95% credible intervals, with grey dashed vertical lines indicating the minimal effect size for significance (0.2 fold-change), red lines denoting non-significant results and blue lines indicating significant ones.

**Figure A2:**
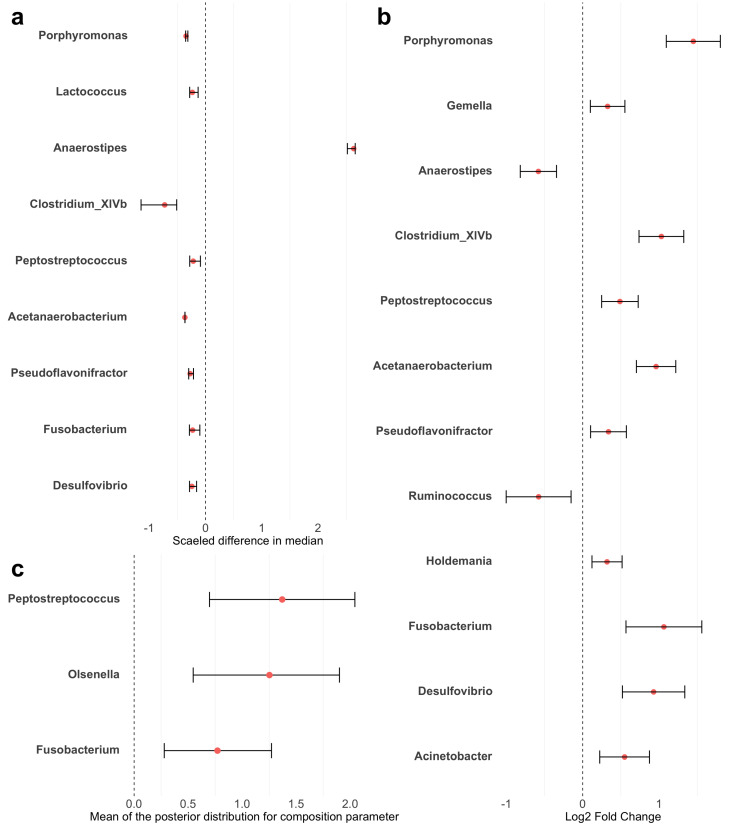
Forest plots of differential taxa abundance between colorectal cancer patients and healthy controls. Red points represent the estimated differences in abundance. Error bars represent the 95% confidence intervals. (**a**) LEfSe: estimates were scaled to standardize the median differences obtained from the Wilcoxon rank sum test. (**b**) ANCOM-BC: estimates are log fold changes. (**c**) sccomp: estimates are the mean of posterior distribution for the composition parameter. LowerCI and UpperCI are the 2.5% and 97.5% quantiles of the posterior distribution for the composition parameter.

LEfSe, ANCOM-BC, and sccomp identified sets of differentially abundant taxa, with some overlap among them (Figure 4a). Detailed forest plots and tables outlining effect sizes, confidence intervals, *p*-values, and adjusted *p*-values are provided in Figure A2 and Table A3, Table A4 and Table A5.

LEfSe (Figure 4c) identified nine differentially abundant taxa between CRC patients and healthy controls: *Pseudoflavonifractor*, *Porphyromonas*, *Peptostreptococcus*, *Lactococcus*, *Fusobacterium*, *Desulfovibrio*, *Clostridium_XIVb*, *Anaerostipes*, and *Acetanaerobacterium*. Effect sizes were quantified using LDA scores, where a positive score indicates enrichment in CRC patients and a negative score indicates depletion. A higher absolute LDA score denotes a greater difference between the two groups. Notably, *Porphyromonas* and *Clostridium_XIVb* are significantly enriched in CRC patients, while *Fusobacterium* and *Anaerostipes* are more abundant in healthy individuals. Among them, *Porphyromonas* and *Fusobacterium* exhibit the most pronounced differences, suggesting a potential role in CRC progression and suppression, respectively.

ANCOM-BC (Figure 4b) identified 12 differentially abundant taxa: *Ruminococcus*, *Pseudoflavonifractor*, *Porphyromonas*, *Peptostreptococcus*, *Holdemania*, *Gemella*, *Fusobacterium*, *Desulfovibrio*, *Clostridium_XIVb*, *Anaerostipes*, *Acinetobacter*, and *Acetanaerobacterium*. Effect sizes were quantified using the log fold change (LFC), where a positive LFC indicates enrichment in CRC patients and a negative LFC indicates depletion. A higher absolute LFC value reflects a greater difference between the groups. Specifically, *Porphyromonas* shows a significant enrichment in CRC patients with an LFC of 1.45, suggesting its abundance is approximately 4.26 times higher than in healthy controls. In contrast, *Ruminococcus* has an LFC of −0.58, indicating its depletion in CRC patients.

**Figure A3:**
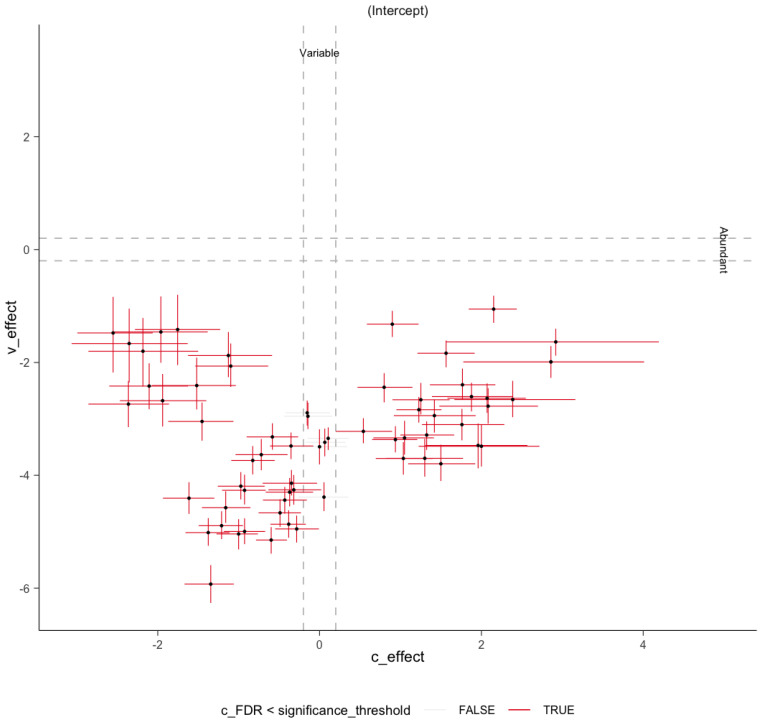
Variability to abundance relationship examined by sccomp. A two-dimensional plot of estimates of composition effect (c_effect) on the *x*-axis and variability effect (v_effect) on the *y*-axis. It shows a positive linear relationship between mean abundance (inverse softmax) and mean variability (log), with bimodality for a group of taxa. Error bars are the 95% credible interval. Red error bars represent significant associations. Gray dashed lines represent the minimum effect size for significance (0.2 fold-change).

**Figure A4:**
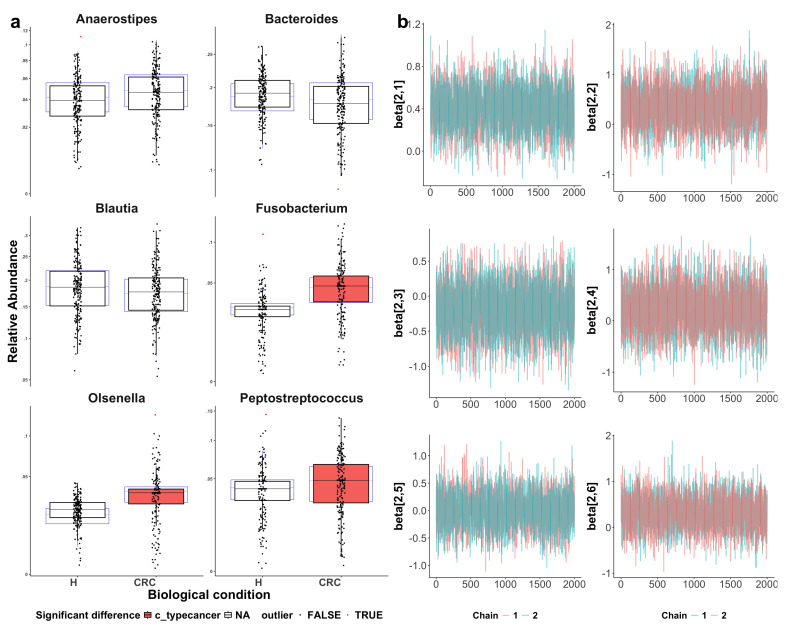
Visualization of sccomp model fit. CRC indicates colorectal cancer patients and H indicates healthy controls. (**a**) Boxplots assessing model adequacy. Blue boxplots represent the predicted data from estimated posterior distributions, while black boxplots indicate the observed data, with outliers highlighted as red triangles. The alignment between the predicted and observed data suggests a good model fit. The three identified differentially abundant taxa (with the CRC group highlighted in red) show more noticeable differences between the CRC and healthy groups as compared to the three reference taxa. (**b**) Visualization of Markov chain Monte Carlo (MCMC) chains from the posterior distribution, used to evaluate parameter convergence and distribution characteristics. The convergence of parameter estimation for *Anaerostipes*, *Bacteroides*, *Blautia*, *Fusobacterium*, *Olsenella*, and *Peptostreptococcus* (from top-left to bottom-right) supports the reliability of the inference.

The sccomp method identified three differentially abundant taxa: *Peptostreptococcus*, *Olsenella*, and *Fusobacterium* (Figure 4d). The differential composition analysis revealed their effect sizes well above the minimum threshold derived from the 95% credible interval, confirming their statistical significance. For reference, *Blautia*, *Bacteroides*, and *Anaerostipes* are also included in the same plot for comparison. Additionally, Figure A3 shows the variability to abundance relationship examined by sccomp. It demonstrates a good fit for a linear association between the mean abundance (inverse softmax) and mean variability (log), with a cluster of taxa showing bimodality. Moreover, Figure A4a presents boxplots of relative abundance of the six taxa (three identified and three reference) in the CRC and control groups, overlaid with boxplots of newly generated data from the fitted posterior distribution. The close alignment between observed and generated data supports a good model fit. Also, as expected, the three identified taxa (with the CRC group highlighted in red) exhibit more noticeable differences between the groups as compared to the three reference taxa. Finally, Figure A4b illustrates the posterior distribution and the convergence of Markov chains, indicating that the estimation has converged, further supporting the reliability of sccomp’s inference.

**Figure A5:**
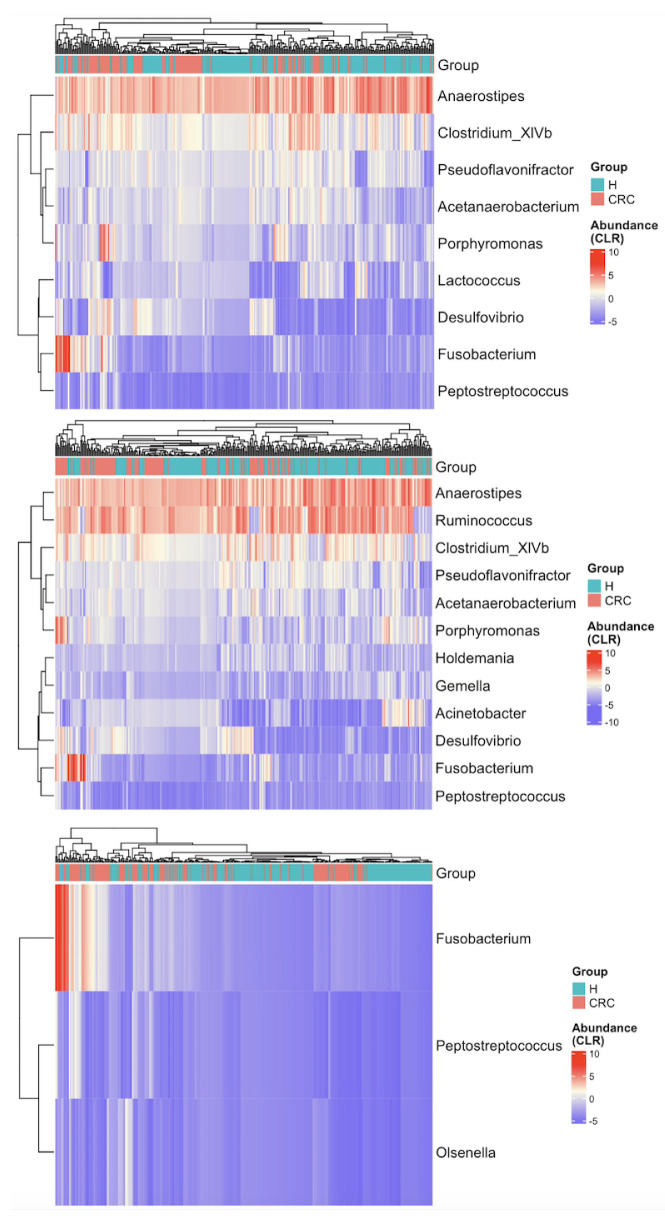
Heatmaps of CLR-transformed abundances of the differentially abundant taxa identified by LEfSe (**top**), ANCOM-BC (**middle**), and sccomp (**bottom**). Each row (*y*-axis) represents a taxon, and each column (*x*-axis) corresponds to a subject. CRC indicates colorectal cancer patients and H indicates healthy controls. The color intensity represents the CLR-transformed abundance of each taxon. Hierarchical clustering of the subjects based on the reduced gut microbial profiles enhances the separation between CRC patients and healthy controls as compared to Figure 3.

Finally, we generated heatmaps using only the 9, 12, and 3 taxa identified by LEfSE, ANCOM-BC, and sccomp, respectively (Figure A5). Compared to Figure 3, all three sets of identified taxa enhance the separation between CRC patients and healthy controls, demonstrating the diagnostic potential of the differentially abundant taxa. Notably, the three taxa identified by sccomp yield the most distinct separation, underscoring the robustness of sccomp’s marker discovery.

## 4. Discussion

In our study, gut microbial richness was significantly higher in CRC patients compared to healthy controls. While some previous studies reported reduced bacterial richness in CRC due to dysbiosis—characterized by the loss of beneficial bacteria, the proliferation of pathogens, or an overall decline in microbial diversity [39,40,41]—others have observed increased richness in CRC patients [42,43], aligning with our findings. Recent research stratifying CRC populations by cancer stage offers a new perspective on microbial richness. These studies indicate that richness tends to increase with CRC stage, with later-stage patients (Stages III and IV) typically exhibiting higher richness than those in earlier stages (Stages I and II) [44]. Regarding gut microbial evenness, some studies have reported a significant decrease in CRC patients, potentially linked to the overgrowth of specific pathogens [45]. However, others, consistent with our findings, found no significant differences in evenness between CRC patients and healthy controls [40]. Studies stratifying CRC patients by stage suggest that evenness follows a similar pattern to richness, with late-stage patients generally displaying higher evenness than early-stage individuals [44]. Since our study did not include data about stages, we were unable to compare gut microbial richness or evenness between stages of CRC. Future studies incorporating detailed staging information are needed to further clarify the interplay between CRC progression and gut microbial diversity. Nevertheless, the confirmed increase in gut microbial richness in this study may serve as an informative biomarker for CRC diagnosis.

To identify differentially abundant taxa, we employed three approaches: LEfSe, ANCOM-BC, and sccomp. Each method has distinct strengths and weaknesses. LEfSe utilized the Wilcoxon rank sum test with effect sizes quantified by LDA. Though widely used with straightforward biological interpretations, it is prone to high false positive rates when applied to highly sparse and complex data. ANCOM-BC corrects for the compositional bias in microbiome data, offering more reliable estimates of differential abundances. It quantifies the difference in abundance using LFC, which is commonly used in omics research to indicate up- or down-regulation. However, it may struggle with extreme sparsity or very small sample sizes, reducing its ability to control FDR. Additionally, when the proportion of differentially abundant taxa exceeds 75%, it may lose the control of FDR. The adapted sccomp model provides a more robust alternative due to three key properties. First, it employs a sum-constrained beta-binomial model with the unimodal mean–variability relationship effectively harnessed. Such a modeling framework can approximate microbiome data more accurately. Second, it incorporates prior information from previously analyzed datasets, leveraging cross-dataset transfer learning to improve its performance in new studies. Third, it iteratively identifies and excludes outliers, enhancing the robustness of its estimations. While this borrowed robust approach may detect fewer signals compared to microbiome-specific methods, it is designed to improve FDR control and enhance the precision of marker discovery.

We conducted a comparative analysis between our study results and existing literature, including the CRC meta-analysis using MicrobiomeHD [14]. Notably, *Fusobacterium* and *Peptostreptococcus* were consistently identified as differentially abundant taxa across ANCOM-BC, LEfSe, and sccomp, aligning with findings from the original MicrobiomeHD meta-analysis. However, it is worth noting that the effect direction of *Fusobacterium* determined by LEfSe was opposite to that by the other two methods and reported in the literature. Specifically, *Fusobacterium nucleatum* is one of the strongest bacteria associated with colorectal carcinogenesis [46], contributing to CRC through mechanisms such as inflammation and immune modulation [47], as well as adhesion and invasion [48]. Similarly, *Peptostreptococcus anaerobius* is a well-established bacterial marker for CRC progression. Studies in animal models have demonstrated that *P. anaerobius* adheres to colorectal mucosa and modulates tumor immunity, creating a microenvironment that favors tumor growth [49,50]. Given their strong associations with CRC, these two bacteria hold potential for early diagnosis and may serve as therapeutic targets. Another taxon identified by sccomp was *Olsenella*. Previous studies have reported its depletion in CRC patients, suggesting a possible protective role in reducing gut inflammation [51]. This aligns with the fact that *Olsenella* belongs to the *Actinobacteria* phylum, some members of which have been implicated in maintaining gut health by regulating inflammatory responses and supporting epithelial barrier function. Although direct evidence regarding *Olsenella*’s role in these processes is limited, it may share similar functions with other *Actinobacteria* members [52]. Notably, our findings indicate the opposite—an increased abundance of *Olsenella* in CRC, highlighting the need for further research into its biological role and functional significance. Furthermore, the detection of enriched taxa such as *Porphyromonas* in our study reinforces their role in CRC pathogenesis [53]. Depleted taxa such as *Anaerostipes* were also identified, some of which produce butyrate, a short-chain fatty acid with anti-inflammatory and anti-carcinogenic properties [54,55].

As functional metagenomic or metabolomic data are not available in the MicrobiomeHD datasets, our study focuses on relative abundance differences of gut bacteria between CRC patients and healthy controls. In the future, the functional data should be incorporated to further analyze pathways of the bacteria, offering deeper insights into the mechanisms underlying CRC pathogenesis. For instance, short-chain fatty acid (SCFA) production, known for its anti-inflammatory and tumor-suppressive properties, has been shown to be altered in CRC patients [56]. Additionally, changes in secondary bile acid metabolism have been implicated in CRC progression, as microbial-derived bile acids can influence intestinal epithelial homeostasis and immune responses [57]. Overall, beyond abundances, integrating functional data will elucidate how the bacteria contribute to CRC development and may uncover novel translational targets.

Beyond the translational value, our findings also hold potential clinical implications for CRC diagnosis, therapeutics, and prognosis. The microbial signatures identified in this study, including diversity metrics and abundances of key bacterial taxa, could serve as non-invasive biomarkers for CRC detection and risk stratification. As gut microbiota profiling becomes more accessible, incorporating microbial-based screening tools with conventional diagnostic methods may enhance early detection accuracy [58,59]. Moreover, the ability of gut microbiota to influence the tumor microenvironment opens new avenues for microbiota-targeted therapies. Approaches such as probiotics, prebiotics, and fecal microbiota transplantation (FMT) are currently being explored as adjunctive treatments for CRC patients [60]. Future clinical trials will be essential to validate the predictive value of microbial markers and assess the efficacy of microbiota-modulating interventions in improving CRC treatment outcomes.

## 5. Conclusions

This study analyzed the gut microbiota of CRC patients and healthy controls using integrated data from various publicly available studies, uncovering differential microbial signatures between the two groups. It identified altered microbial diversities, as well as taxa that were significantly enriched or depleted in CRC patients, providing valuable insights into the role of gut microbiota in both promoting and inhibiting CRC progression. Biologically, the findings enhance our understanding of the gut microbiota’s contributions to the development and suppression of CRC. Particularly, the differentially abundant taxa have the potential to transform the early detection, therapeutics, and prognosis evaluation of CRC, paving the way for improved disease management and treatment. Methodologically, this study showcases the effectiveness of adapting robust methods from single-cell research to enhance the precision of microbial marker discovery.

## Data Availability

The MicrobiomeHD database is publicly available at https://zenodo.org/records/840333, accessed on 3 March 2025. Reporting of the five colorectal cancer case-control studies complies with the STROBE case-control study checklist (EQUATOR Network). The code to reproduce the analysis can be found at https://github.com/hanhuiyeye/RobustLearning-GutMicrobiome-CRC, accessed on 3 March 2025.

## Appendix A

**Table A1:** Summary of the five colorectal cancer studies from the MicrobiomeHD database.

Study	Cases ^1^	Controls ^1^	Region	Sequencer	16S Region
Baxter (2016) [22]	120	172	USA	Miseq	V4
Chen (2012) [23]	21	22	China	454	V1–V3
Wang (2012) [24]	44	54	China	454	V3
Zeller (2014) [26]	41	75	France	Miseq	V4
Zackular (2014) [25]	30	30	USA	Miseq	V4

^1^ The numbers shown in this table correspond to the processed datasets through the MicrobiomeHD pipeline, which may differ from the values reported in the original studies.

**Table A2:** Summary of differential abundance analysis methods: LEfSe, ANCOM-BC, and sccomp.

Method	Model Formula	Key Features
LEfSe	Compare taxon abundance between healthy and diseased individuals	- Linear discriminant analysis for effect size estimation - Non-parametric tests (Kruskal–Wallis/Wilcoxon rank sum)
ANCOM-BC	$E\left[\log\left(Y_{ijk}\right)\right]=d_{ik}+\mu_{jk}$ - $Y_{ijk}$: Count of taxon *j* in individual *i* from group *k* (k=0: healthy; k=1: diseased) - $d_{ik}$: Effect of sampling fraction for individual *i* from group *k* - $\mu_{jk}$: Effect of true count of taxon *j* for an individual from group *k*	- Corrects bias introduced by differences in sampling fractions - Provides statistically valid test - Provides confidence intervals for effect size of each taxon - Controls false discovery rate and maintains adequate power
sccomp	$E\left[Y_{ij}\right]=\gamma_{0j}+\gamma_{1j}\cdot {\mathrm{DiseaseState}}_{i}$ - $Y_{ij}$: Count of taxon *j* in individual *i* - $\gamma_{0j}$: Baseline count (healthy) - $\gamma_{1j}$: Effect of disease on count of taxon *j*	- $Y_{ij}$ follows a sum-constrained beta-binomial distribution - Estimates by Hamiltonian Monte Carlo via Bayesian inference - Captures mean—variability relationship of microbial abundances - Developed for single-cell and adapted for microbiome data

**Table A3:** Estimates of differential taxa abundance by LEfSe.

Taxa	Estimate ^1^	LowerCI	UpperCI	*p*-Value ^2^	*q*-Value ^3^
*Porphyromonas*	−0.346	−0.353	−0.313	<0.001	<0.001
*Lactococcus*	−0.233	−0.281	−0.132	0.001	0.006
*Anaerostipes*	2.633	2.520	2.659	<0.001	<0.001
*Clostridium_XlVb*	−0.725	−1.145	−0.512	<0.001	<0.001
*Peptostreptococcus*	−0.218	−0.280	−0.091	<0.001	<0.001
*Acetanaerobacterium*	−0.367	−0.366	−0.366	<0.001	<0.001
*Pseudoflavonifractor*	−0.273	−0.299	−0.214	0.001	0.003
*Fusobacterium*	−0.229	−0.286	−0.101	<0.001	<0.001
*Desulfovibrio*	−0.242	−0.284	−0.159	<0.001	<0.001

^1^ Estimates were scaled to standardize the median differences obtained from the Wilcoxon rank sum test. ^2^ *p*-values were calculated using the Wilcoxon rank sum test. ^3^ The *p*-values were adjusted using Benjamini–Hochberg correction.

**Table A4:** Estimates of differential taxa abundance by ANCOM-BC.

Taxa	Estimate ^1^	LowerCI	UpperCI	*p*-Value ^2^	*q*-Value ^3^
*Porphyromonas*	1.449	1.095	1.803	<0.001	<0.001
*Gemella*	0.326	0.100	0.553	0.004	0.024
*Anaerostipes*	−0.581	−0.818	−0.343	<0.001	<0.001
*Clostridium_XlVb*	1.031	0.737	1.324	<0.001	<0.001
*Peptostreptococcus*	0.487	0.248	0.726	<0.001	<0.001
*Acetanaerobacterium*	0.961	0.703	1.219	<0.001	<0.001
*Pseudoflavonifractor*	0.338	0.103	0.572	0.005	0.024
*Ruminococcus*	−0.577	−1.002	−0.152	0.008	0.036
*Holdemania*	0.319	0.121	0.516	0.002	0.010
*Fusobacterium*	1.063	0.567	1.560	<0.001	<0.001
*Desulfovibrio*	0.929	0.520	1.337	<0.001	<0.001
*Acinetobacter*	0.549	0.223	0.874	0.001	0.007

^1^ Estimates is derived from the log fold change. ^2^ The *p*-values were reported by ANCOM-BC. ^3^ The *p*-values were adjusted using Benjamini–Hochberg correction.

**Table A5:** Estimates of differential taxa abundance by sccomp.

Taxa	Estimate ^1^	LowerCI ^2^	UpperCI ^2^	*p*-Value ^3^	*q*-Value ^4^
*Fusobacterium*	0.772	0.279	1.272	0.014	0.006
*Olsenella*	1.253	0.547	1.901	0.002	0.003
*Peptostreptococcus*	1.371	0.698	2.044	0.001	0.002

^1^ Estimates are the mean of posterior distribution for the composition parameter. ^2^ LowerCI and UpperCI are the 2.5% and 97.5% quantiles of the posterior distribution for the composition parameter. ^3^ The *p*-values represent the probability of the null hypothesis (no difference) for a composition. ^4^ The *q*-values indicate the false discovery rate of the null hypothesis (no difference) for a composition.
